# Climate change and tropical medicine in the Amazon: contributions from a research center, gaps and future priorities

**DOI:** 10.1590/0037-8682-0470-2025

**Published:** 2026-06-22

**Authors:** Fernando Almeida-Val, Eduardo Fernandes, Alexandre Vilhena Silva-Neto, Bernardo Maia Silva, Felipe Nery Saldanha Braga, Erika de Oliveira Gomes, Daniel Barros de Castro, Camila Fabbri, Luarle de Souza Lima, Felipe Leão Gomes Murta, Rubelmar Maia de Azevedo Cruz-Neto, Taynna Vernalha Rocha Almeida, Vanderson de Souza Sampaio, Guilherme Peixoto Tinôco Arêas, Wuelton Marcelo Monteiro

**Affiliations:** 1 Fundação de Medicina Tropical Doutor Heitor Vieira Dourado, Manaus, AM, Brasil.; 2 Universidade do Estado do Amazonas, Manaus, AM, Brasil.; 3 Universidade Nilton Lins, Manaus, AM, Brasil.; 4 Universidade Federal do Amazonas, Manaus, AM, Brasil.; 5 Instituto Nacional de Pesquisas na Amazônia, Manaus, AM, Brasil.; 6 Fundação Oswaldo Cruz Amazônia, Instituto Leônidas & Maria Deane, Manaus, AM, Brasil.; 7 Ministério da Saúde, Brasília, DF, Brasil.; 8 Instituto Todos pela Saúde, São Paulo, SP, Brasil.

**Keywords:** Climate change, Tropical medicine, Communicable diseases, Public health, Vector borne diseases

## Abstract

The Amazon Basin is undergoing rapid climatic and environmental changes, with direct impacts on infectious disease transmission and public health. Rising temperatures, altered rainfall patterns, and more frequent extreme events are reshaping vector and pathogen dynamics. Additionally, deforestation and land-use change increase human and animal exposure. These combined factors intensify the risk of disease transmission in the region. This narrative review synthesizes evidence generated by researchers from a reference center in tropical medicine in the Amazon, integrating epidemiological analyses, experimental studies, ecological and predictive modelling, and health systems research conducted over the past decade. The evidence indicates that temperature and hydrological variability affect vector competence, pathogen development, and host-vector contact rates, producing nonlinear and context-dependent transmission outcomes. Extreme hydroclimatic events additionally disrupt healthcare delivery, hinder surveillance, and compromise continuity of care for infections requiring sustained treatment, such as tuberculosis and HIV. Emerging advances, including species distribution modelling, remote sensing, and integrated climate-epidemiological surveillance, provide predictive capacity to anticipate outbreaks and identify emerging hotspots. However, substantial research gaps persist, particularly regarding multi-stressor interactions, mechanistic pathways of climate-pathogen-vector adaptation, and health system resilience under climatic extremes. From the perspective of a reference center that integrates education, research, and healthcare delivery in tropical medicine, this article articulates a research agenda for climate-informed tropical medicine, highlighting priorities for interdisciplinary research, surveillance innovation, and adaptive public health strategies in the Amazon and other climate-sensitive regions.

## INTRODUCTION

The Amazon Basin, the world’s largest tropical rainforest, is a biodiversity hotspot and a key regulator of the global climate. Spanning over 6 million km² across nine countries, it plays essential roles in carbon cycling and hydrological regulation. However, rising temperatures, altered rainfall, extreme climatic events, and deforestation are rapidly transforming its landscapes. These changes threaten both ecosystems and human health^1^ ^,^ ^2^.

These changes profoundly affect disease dynamics. Temperature and precipitation shift influence vector survival and pathogen development, while altered hydrology impacts breeding sites. Land-use change, particularly deforestation, increases human contact with sylvatic cycles and facilitates vector expansion into peri-urban areas^3^ ^,^ ^4^. Climate variability also drives zoonotic, water-borne, and food-borne diseases; El Niño affects leishmaniasis incidence^5^, and hydrological changes modulate malaria and leptospirosis^6^. Socio-environmental vulnerabilities intensify these effects^7^.

**FIGURE 1:**
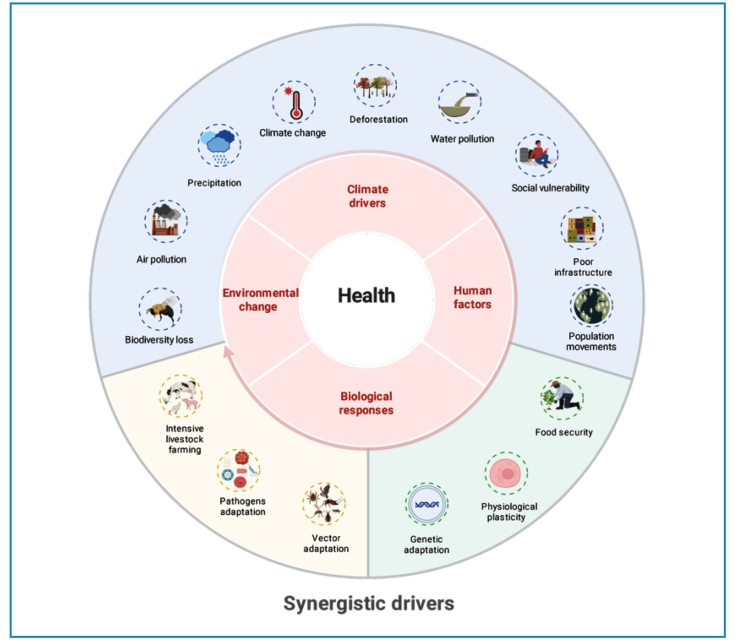
Synergistic Drivers of Climate-Sensitive Health. **Caption:** Climatic variability, land-use change, biodiversity loss, and socio-environmental vulnerabilities converge to shape the distribution, intensity, and outcomes of climate-sensitive diseases. Understanding these interconnected processes - and how they operate within the unique socio-ecological context of the Amazon - is essential to guide research priorities, strengthen adaptive capacity, and inform future public health strategies. Created in https://BioRender.com.

The 2024 drought, the most severe in decades, caused record-low river levels, disrupting healthcare delivery and exposing fragile health logistics. Severe wildfires and particulate pollution worsened respiratory disease^8^. Key drivers of climate-sensitive diseases are summarized in Figure 1. Understanding these interactions within the scope of research conducted in the Brazilian Amazon is critical to anticipate future threats. This review synthesizes evidence generated by a reference center in tropical medicine in the Brazilian Amazon and its collaborators, identifies research gaps, and proposes priorities for surveillance, prevention, and adaptation in climate-sensitive contexts. 

## METHODS AND SCOPE OF THE REVIEW

This article adopts a narrative review and institutional perspective to synthesize climate-health research conducted by a tropical medicine reference center in the Brazilian Amazon and its collaborating institutions over the past decade (2014-2024). The scope includes peer-reviewed articles, preprints, and ongoing analyses produced within this research network, encompassing epidemiological investigations, experimental and laboratory studies, ecological and predictive modelling, health systems research, and participatory approaches. Examples discussed throughout the manuscript are illustrative of broader thematic research lines rather than an exhaustive catalog of all studies conducted by the center.

Studies were identified through internal institutional records, project databases, and systematic tracking of publications and preprints authored by researchers affiliated with the center and long-term collaborators. The review is not intended as an exhaustive systematic review of all climate-health research in the Amazon, but rather as a structured synthesis of representative and thematically coherent evidence generated within this institutional program.

The emphasis on specific conditions - including malaria, arboviral diseases, leishmaniasis, snakebite envenomation, tuberculosis, and HIV - reflects both the historical mandate of the center in tropical medicine and the relative maturity of these research lines. Other climate-sensitive outcomes (e.g., heat-related illness, wildfire smoke exposure, mental health, and nutrition) are discussed where evidence from this research program is emerging or where clear gaps remain, and are highlighted as priorities for future investigation.

## ADVANCES IN UNDERSTANDING CLIMATE-DISEASE DYNAMICS FROM A TROPICAL MEDICINE REFERENCE CENTER IN THE AMAZON

### Vector ecology and environmental change

The ecology and behavior of disease vectors in the Amazon are strongly shaped by climatic conditions, environmental changes, and anthropogenic pressures. Within this research program, epidemiological and experimental studies have examined how temperature, humidity, and rainfall influence key aspects of vector life cycles - including development, reproduction, survival, and competence - thereby modulating transmission dynamics. Hendy et al. (2020)^9^ showed that vertical stratification of arbovirus vectors such as *Haemagogus* and *Sabethes* varies significantly across forest canopy layers, driven by microclimatic gradients. These findings from studies conducted in the central Brazilian Amazon demonstrate that even subtle climatic fluctuations can alter mosquito niches, potentially reshaping the interface between sylvatic and urban transmission cycles.

Field evidence from forest edges near Manaus further highlights how microclimate and rainfall pulses shape vector distribution. Hendy et al. (2021)^10^ observed that *Haemagogus janthinomys* was more abundant at 9 m during hotter, drier afternoons when vertical gradients were strongest, while *Psorophora amazonica* showed no clear stratification. Species occurrence was associated with recent precipitation, and *Hg. janthinomys* was most frequently collected around 29.9°C. These results provide mechanistic insight within the geographic scope of these investigations into how short-term hydroclimatic variability and canopy-ground thermal structure influence human-wildlife contact potential.

Surveillance data reinforce the permeability of ecological boundaries, as Gomes et al. (2023)^11^ reported Zika virus in *Aedes albopictus* from urban forest fragments in Manaus, highlighting the role of bridge vectors in pathogen transmission in peri-urban Amazonian environments.

Experimental studies indicate that climate change can affect vector physiology and competence. Under IPCC-simulated conditions, *Anopheles aquasalis* showed reduced size and colony collapse under extreme warming, while environmental stress altered survival and immune gene expression without changing *P. vivax* infection rates. These findings suggest that climate impacts transmission not only through ecological changes but also via physiological mechanisms affecting vector competence^12^ ^,^ ^13^.

Finally, climate-induced shifts in vegetation structure and forest edge dynamics - often linked to deforestation and land-use change - create new habitats and alter host-seeking behavior, increasing human exposure in peri-urban and frontier areas. Together, these examples illustrate key lines of investigation pursued within this center’s climate-health agenda rather than an exhaustive synthesis of all vector-climate studies conducted in the Amazon.

### Spatio-temporal patterns and disease distribution

Climatic variability is a primary driver of spatio-temporal changes in disease incidence across the Amazon. Analyses conducted within this research network have examined how rainfall, temperature, and humidity govern vector proliferation, pathogen development, and host-vector contact, shaping seasonal and interannual disease trends. This is particularly evident in the case of leishmaniasis, a disease that is highly sensitive to environmental fluctuations. Chagas et al. (2022)^14^ showed that ATL incidence peaks during the rainy season and is positively associated with temperature and humidity, highlighting climatic seasonality as a key driver of transmission and the need to incorporate environmental variables into predictive models and control strategies.

Land-use change further complicates these dynamics. Rodrigues et al. (2018)^15^ showed that ATL risk varies with the timing of deforestation. Areas with recent forest clearance had significantly higher incidence than areas deforested decades earlier. This likely reflects increased human-vector contact in transitional landscapes created by agricultural expansion and fragmentation, which favor sandflies and human activity. In contrast, long-term deforestation may reduce risk by degrading conditions needed for vector survival. Overall, the findings highlight a non-linear, context-dependent relationship between land-use history and ATL transmission.

**FIGURE 2:**
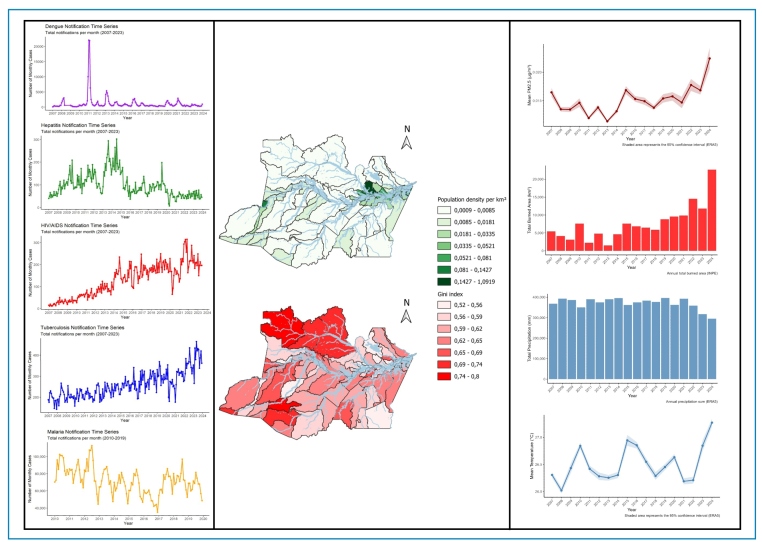
Spatio-temporal dynamics of climate, environmental change, socio-demographic factors, and disease burden in the Amazon Basin. **Caption:** Spatio-temporal dynamics of climate, socio-environmental factors, and infectious disease burden in the Brazilian Amazon. Temporal trends of tuberculosis, malaria, HIV/AIDS, hepatitis, and dengue were derived from SINAN/Datasus and Ministry of Health databases, representing annual reported cases in Amazonas between 2007 and 2023 (2012-2024 for malaria). Climatic variables (precipitation and temperature) were calculated from monthly municipal averages based on Copernicus ERA5 Reanalysis, with accumulated annual precipitation and 95% confidence intervals shown. Burned area data were obtained from INPE as annual totals from summed monthly records. Population density maps represent the mean annual density across 2007-2024, while Gini coefficients reflect socioeconomic inequality based on IBGE 2010 data. Together, these layers illustrate how environmental variability, demographic distribution, and social inequality intersect with infectious disease dynamics over time and space in the Amazon Basin.

These spatio-temporal patterns show that climate variability and land-use change intersect to shape the geography of infectious diseases in the Amazon. Seasonal shifts in temperature and precipitation influence vector dynamics and pathogen development, while deforestation and habitat fragmentation create new ecological interfaces that heighten human exposure. However, disease distribution is not driven by biophysical factors alone - it is also shaped by socio-economic and infrastructural conditions that mediate vulnerability and adaptive capacity. Understanding these dimensions is essential to interpret transmission patterns and design effective, context-specific interventions. Spatio-temporal dynamics documented by surveillance and research activities of this reference center are illustrated in Figure 2.

While the studies highlighted here reflect research lines developed within this institutional network, the broader literature on malaria and other climate-sensitive diseases in the Amazon documents more heterogeneous and stage-specific dynamics, particularly in frontier settings. Empirical and modelling studies have shown that malaria risk varies across phases of frontier expansion and is strongly mediated by population mobility, migration flows, and occupational patterns. Climatic responses are also non-linear and spatially heterogeneous across sub-regions of the basin. The examples presented in this section therefore illustrate specific analytical approaches developed within this research program rather than exhaustively capturing the full complexity of climate-environment-health interactions across the Amazon Basin.

### Socio-environmental determinants and climate-sensitive health outcomes

Climatic and ecological processes interact with socio-environmental determinants to shape disease risk and outcomes. Within this research program, investigations have explored how geographic isolation, socio-economic inequality, and uneven access to health services - all of which amplify the health impacts of climate variability. Socioeconomic status, occupational exposure, and infrastructure deficits mediate how communities experience and respond to climate-sensitive diseases. Castro et al. (2018)^16^ showed that socio-environmental factors such as housing, sanitation, and income are strongly associated with dengue epidemics in the Amazon, highlighting how social vulnerability interacts with climate to shape disease risk^16^.

Human exposure is strongly shaped by land use and economic activities, with agriculture and extractive work increasing contact with vectors. Migration and urbanization further alter disease distribution by changing settlement patterns and access to care. Addressing these drivers requires integrated strategies combining climate adaptation, poverty reduction, infrastructure, and health system strengthening. Land-use transformation is a consistent driver of disease emergence, linking anthropogenic environmental change to shifting transmission patterns. Terrazas et al. (2015)^17^ showed that deforestation increases malaria risk by altering habitats and enhancing human-mosquito contact. Recently, Kalbus et al. (2021)^18^ linked deforestation and urban expansion to increased dengue incidence, highlighting the role of landscape fragmentation in vector dynamics. Together, these findings show that land-use change is a key driver of climate-sensitive diseases, interacting with socio-economic factors and amplifying transmission risk.

Climatic shifts also fundamentally alter the biological and ecological conditions underlying disease transmission. Changes in temperature, rainfall, humidity, and wind patterns can directly influence vector abundance, survival, and feeding behavior, as well as pathogen development. 

Cella et al. (2019)^19^ showed that moderate temperature increases accelerate *Plasmodium* spp. development and shorten the extrinsic incubation period, potentially expanding malaria transmission, while extreme heat reduces mosquito survival. These findings highlight the non-linear effects of climate and, combined with social and environmental factors, reinforce the need for predictive models that integrate climatic, ecological, and socio-economic dimensions.

Beyond environmental and ecological determinants, vulnerability is also shaped by behavioral, cultural, and social factors influencing exposure, prevention practices, and access to care. Incorporating qualitative and participatory approaches is therefore critical to complement quantitative evidence and guide future research agendas aimed at deepening our understanding of how communities perceive, experience, and adapt to climate-sensitive health risks.

Such qualitative perspectives are particularly valuable in revealing how these processes unfold locally. Community-based evidence from riverine populations underscores how climatic seasonality interacts with geographic isolation and infrastructural gaps to shape health outcomes. In Tabatinga (Western Brazilian Amazon), Santos et al. (2025)^20^ reported that severe droughts and floods exacerbate food and water insecurity, increase gastrointestinal illnesses and injuries, and restrict access to healthcare. These findings highlight the role of social and infrastructural vulnerability in climate-sensitive disease burdens and show that hydroclimatic extremes can rapidly intensify vulnerabilities and reshape exposure patterns in the Amazon.

### Extreme events and neglected health risks

Beyond gradual climate trends, extreme weather events have been examined within this research program as critical triggers of disease emergence and spread in the Amazon. These events disrupt ecosystems, alter vector habitats, and intensify human exposure, often leading to unexpected outbreaks. One striking example is the relationship between precipitation and snakebite envenomation, a neglected but significant public health problem. Alcântara et al. (2018)^21^ demonstrated that the incidence of *Bothrops* snakebites is closely associated with rainfall dynamics, with pronounced peaks during the wet season when snake activity increases and agricultural labor intensifies. 

Climatic modulation of snakebite risk is further supported by evidence linking hydrological changes to both ecological and social determinants. Santos et al. (2019)^22^ showed that seasonal flooding and environmental variability drive snakebite incidence by influencing snake distribution and human exposure. These effects are intensified in remote areas, where limited healthcare access exacerbates disease burden and existing inequalities.

While the direct effects of extreme events are increasingly recognized, their indirect impacts on health are equally critical. Hydroclimatic disruptions strain healthcare systems, interrupt services, and undermine control programs, particularly affecting diseases that require continuous care, such as tuberculosis and HIV.

Long-term surveillance data illustrate how such extremes disrupt infection management in the Amazon. In a recent preprint, Menezes et al.^23^ analyzed 24 years of data and showed that severe droughts substantially impaired tuberculosis and HIV care cascades across all 62 municipalities. The 2015-2016 drought was linked to sharp increases in treatment abandonment and disease-specific mortality, especially among men, while the 2023-2024 drought - despite unprecedented mitigation efforts - still revealed persistent gaps in adherence and survival. These findings, although based on preliminary analyses pending peer review, demonstrate how environmental shocks undermine continuity of care, amplify gendered vulnerabilities, and expose structural weaknesses in the health system, underscoring the need for anticipatory adaptation strategies that integrate climate forecasts and decentralized treatment to strengthen resilience.

Human fascioliasis offers another example of a neglected disease closely linked to climatic and environmental conditions. A review by Maciel et al. (2023)^24^ identified 66 confirmed cases of fascioliasis in Brazil between 1958 and 2022, under a wide range of climatic conditions This broad ecological tolerance reflects the dependence of the parasite and its intermediate snail hosts on environmental conditions and suggests that fascioliasis could expand into new regions as temperature and rainfall regimes shift. These patterns underscore the need to include water- and food-borne zoonoses in discussions of climate-sensitive diseases and anticipate their potential future spread under changing scenarios.

### Predictive modelling and surveillance innovations

Regional-scale species distribution modelling applied in studies conducted by this research network has clarified how climate, land use, and demography may reshape malaria risk and vector niches in northern South America. Using Maxent with presence-only data and 23 predictors (19 bioclimatic variables plus topography, hydrology, land-use/land-cover, and population), Alimi et al. (2015)^25^ projected current and future suitability of malaria and its vectors to 2050 and 2070 under IPCC AR5 scenarios. They found a contraction of malaria-suitable areas by ~6-17% by 2070, despite modest vector range expansion, with elevation, precipitation, and temperature as dominant predictors. These results argue for climate-land-use-demography-integrated early-warning systems that anticipate shifting vector frontiers while sustaining elimination planning where suitability is projected to decline.

A major step forward has been the integration of epidemiological surveillance with environmental and climatic data, enabling more proactive responses. Sironi et al. (2018)^26^ developed an integrated database linking malaria surveillance systems with environmental and socio-demographic data across the Amazon (2010-2015). This framework supports ecological analyses, machine learning-based parasite classification, and epidemic forecasting. By breaking down data silos and combining diverse streams, integrated surveillance systems can improve early warning capabilities and guide targeted interventions.

Advances in spatial modelling and machine learning enhance prediction by integrating climate, land use, and socio-demographic factors. These tools are used to map hotspots, estimate risk, and guide resource allocation, supporting early warning systems adapted to Amazonian conditions. As data and analytical capacity expand, predictive modelling is becoming central to adaptive public health strategies.

Recent methodological developments illustrate this potential. New modelling frameworks combining high-resolution climatic data, hydrological dynamics, and surveillance records have improved predictive performance, enabling earlier outbreak detection and more precise spatial risk mapping. Such integrative models show that coupling environmental variability with surveillance enhances forecasting accuracy, supports proactive vector control, and strengthens preparedness under accelerating climate change^27^. Together, these examples represent methodological lines developed within this center and illustrate emerging directions for climate-health modelling, rather than a comprehensive inventory of predictive efforts across the Amazon.

## ONGOING RESEARCH AND HUMAN CAPACITY BUILDING

The increasing complexity of climate-health interactions in the Amazon has fostered a dynamic research agenda within this reference center, integrating fieldwork, laboratory experimentation, epidemiological analysis, predictive modelling, and participatory science. Numerous projects address complementary dimensions to inform adaptive health policies and strategies. Within vector ecology and eco-epidemiology, experiments assess how future climate scenarios affect vector life cycles, competence, and immune responses. Other studies examine how meteorological variables, land use, and sociodemographic factors shape snakebite incidence, expanding knowledge on neglected climate-sensitive diseases.

Secondary data and time-series modeling are being used to estimate future disease burden. Investigations on long-term trends in tuberculosis and HIV linked to climatic extremes, while complementary analyses evaluate how extreme events affect emergency care (SAMU), generating insights to strengthen system resilience. Participatory initiatives, such as the “*Vozes Ribeirinhas*” project, develop community-led surveillance integrating traditional knowledge with early-warning tools. The *VISAmazônia* platform is combining climatic, environmental, and epidemiological data with AI for predictive surveillance and investigates climate impacts on respiratory, cardiovascular, infectious, and mental health. 

This expanding landscape also drives capacity building. Graduate projects and fellowships train a new generation of scientists, while original datasets, publications, and technological tools strengthen the evidence base for policy and adaptation strategies, positioning the Amazon as a living laboratory for climate-health research.

## RESEARCH GAPS AND FUTURE AGENDA

### Mechanistic gaps: Pathogen-vector-host interactions

Despite significant advances generated by research conducted in the Brazilian Amazon, important gaps remain in understanding how climate change shapes the distribution and burden of tropical diseases, particularly regarding the mechanistic pathways underlying these processes. Most studies have focused on vector-borne diseases such as malaria, arboviruses, and leishmaniasis, leaving helminth infections, water- and food-borne pathogens, zoonotic spillovers, environmentally linked chronic infections, and associated sequelae, largely unexplored. Few investigations have addressed the synergistic impacts of multiple drivers - including climate variability, land-use change, deforestation, and socio-economic inequality - on transmission dynamics.

Crucially, our knowledge of how environmental shifts influence pathogen-vector-host relationships at molecular, physiological, and ecological levels remains fragmentary. While experimental data demonstrate that temperature and CO₂ can affect vector competence and immune responses, integrated evidence linking these processes to pathogen development and population-level transmission is scarce. Future research should combine controlled experiments, field ecology, and multi-omics approaches to uncover adaptive responses and potential evolutionary feedback under changing climates.

### Advances in predictive modelling and surveillance

Predictive models for climate-sensitive diseases in the Amazon remain constrained by incomplete datasets, low spatial resolution, and simplified assumptions that overlook non-linear dynamics, feedback, and multi-pathogen interactions. Overcoming these limitations requires integrating high-resolution climatic, ecological, epidemiological, and socio-economic data into dynamic forecasting frameworks, complemented by clinical records and healthcare system indicators to strengthen early-warning capacity. Advanced analytical approaches - including machine learning, graph neural networks, and hybrid models - can enhance risk prediction, anticipate hotspots, and support proactive public health responses when linked to real-time environmental monitoring and surveillance data.

Future predictive frameworks should also account for hierarchical and temporal dependencies intrinsic to Amazonian systems. Hierarchical mixed-effects models, both linear and nonlinear, provide robust and interpretable estimates of spatial heterogeneity and temporal variability, capturing regional trends and local deviations under diverse climatic and socio-environmental conditions. In parallel, Recurrent Neural Networks- particularly Long Short-Term Memory architectures - can retain long-term temporal signals^28^ and capture complex feedback in disease dynamics influenced by climatic oscillations or extreme events, improving the modelling of delayed or cumulative impacts. Although deep learning improves predictive accuracy, mixed-effects models remain essential for interpretability, while Monte Carlo simulations enable scenario exploration and uncertainty estimation. Together, these approaches enhance the robustness and policy relevance of predictive modelling for climate-adapted health strategies in the Amazon.

### Health system resilience and adaptive capacity

The interaction between climate change and health system performance remains a critical but underexplored dimension of climate-health research in the Amazon. Hydroclimatic extremes - such as severe droughts, prolonged floods, and heatwaves - routinely disrupt service delivery, delay emergency response, and compromise surveillance systems. These disruptions have far-reaching consequences: interruptions in transport routes impede the distribution of medicines and vaccines, damage to infrastructure delays care provision, and prolonged isolation limits access to essential services in remote and riverine areas. Such events reveal deep structural vulnerabilities and underscore the need to integrate climate risk into health planning and operations.

Building adaptive capacity requires investments in resilient infrastructure, flexible logistics, and anticipatory planning. Health systems should integrate climate indicators, strengthen supply networks, and adopt alternative care models suited to variable hydrological conditions. Expanding telemedicine and mobile services, along with integrated early-warning systems, can ensure continuity of care and improve outbreak prediction and response.

### Community engagement

Co-designing surveillance, communication, and intervention strategies with Indigenous, traditional, and riverine populations enhances trust, accelerates detection, and ensures culturally appropriate adaptation measures. Health workers, locally managed reporting networks, and participatory monitoring strengthen the interface between local knowledge and formal health systems, improving operational relevance and resilience. Embedding these practices into routine care and emergency preparedness enhances adaptive capacity, ensures more equitable access to services, and builds the foundations for health systems capable of proactively responding to the accelerating impacts of climate change.

**FIGURE 3:**
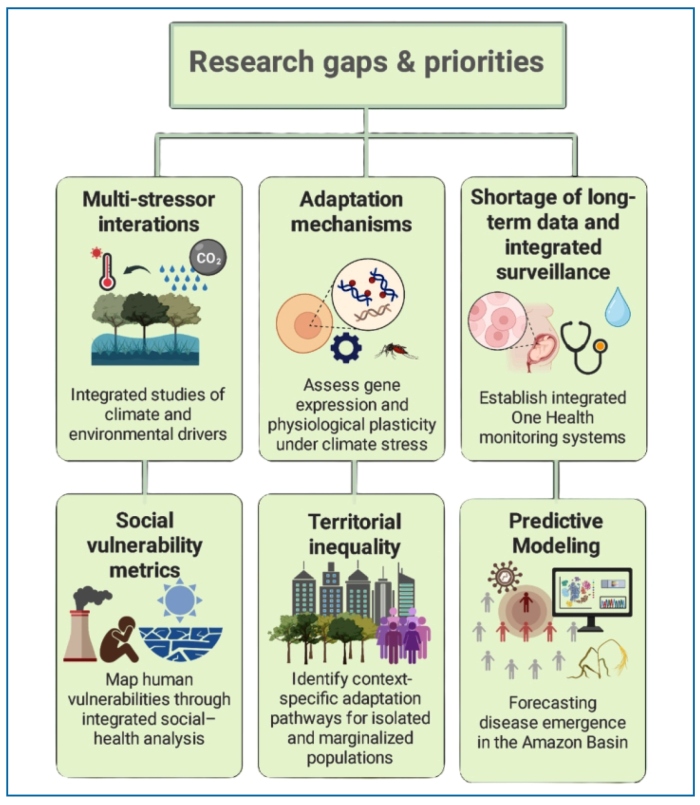
Research gaps and proposed priorities for climate-health research in the Amazon. **Caption:** Key frontiers include understanding multi-stressor interactions, uncovering molecular and physiological adaptation mechanisms, and developing long-term, integrated surveillance systems. Advancing social vulnerability metrics, territorial equity analyses, and predictive modeling will be essential to anticipate disease emergence, guide interventions, and design adaptation strategies tailored to the complex socio-ecological realities of the Amazon Basin. Created in https://BioRender.com.

Collectively, these elements underscore that strengthening health system resilience is not only a response to current climate pressures but also a prerequisite for future preparedness. This integrated perspective provides a foundation for advancing climate-health research and guiding adaptation strategies tailored to Amazonian realities. Research priorities are outlined in Figure 3.

## CONCLUSIONS

Evidence generated by this tropical medicine reference center in the Amazon shows that climate change is transforming the landscape of infectious and climate-sensitive diseases, influencing not only vector ecology and transmission dynamics but also social vulnerability, healthcare access, and system resilience. Evidence indicates that even subtle environmental shifts can modify pathogen-vector-host interactions, while extreme events and land-use changes intensify exposure and inequities. However, significant knowledge gaps remain, concerning multi-stressor interactions, mechanistic pathways, and the effectiveness of adaptive strategies. Addressing these challenges requires integrative approaches that link climatic, ecological, epidemiological, and socio-economic perspectives, alongside strengthened predictive modeling, interdisciplinary collaboration, and the inclusion of Indigenous and local knowledge. Incorporating climate considerations into surveillance, prevention, and healthcare planning is crucial to improve preparedness, response, and recovery, guiding evidence-based public health actions to mitigate climate impacts in the Amazon.

## Data Availability

Research data is only available upon reasonable request.
